# CD154 Costimulation Shifts the Local T-Cell Receptor Repertoire Not Only During Thymic Selection but Also During Peripheral T-Dependent Humoral Immune Responses

**DOI:** 10.3389/fimmu.2018.01019

**Published:** 2018-05-17

**Authors:** Anke Fähnrich, Sebastian Klein, Arnauld Sergé, Christin Nyhoegen, Sabrina Kombrink, Steffen Möller, Karsten Keller, Jürgen Westermann, Kathrin Kalies

**Affiliations:** ^1^Institute of Anatomy, University of Luebeck, Luebeck, Germany; ^2^Centre de Recherche en Cancérologie de Marseille (CRCM) U1068 INSERM – UMR7258 CNRS – Institut Paoli Calmette, Aix-Marseille University, UM105, Marseille, France; ^3^Institute of Mathematics, University of Luebeck, Luebeck, Germany; ^4^Institute for Biostatistics and Informatics in Medicine and Ageing Research, Rostock, Germany

**Keywords:** CD154 costimulation, T-cell repertoire, humoral immune response, sheep red blood cells, spleen, T:B-cell interaction

## Abstract

CD154 is a transmembrane cytokine expressed transiently on activated CD4 T cells upon T-cell receptor (TCR) stimulation that interacts with CD40 on antigen-presenting cells. The signaling via CD154:CD40 is essential for B-cell maturation and germinal center formation and also for the final differentiation of CD4 T cells during T-dependent humoral immune responses. Recent data demonstrate that CD154 is critically involved in the selection of T-cell clones during the negative selection process in the thymus. Whether CD154 signaling influences the TCR repertoire during peripheral T-dependent humoral immune responses has not yet been elucidated. To find out, we used CD154-deficient mice and assessed the global TCRβ repertoire in T-cell zones (TCZ) of spleens by high-throughput sequencing after induction of a Th2 response to the multiepitopic antigen sheep red blood cells. Qualitative and quantitative comparison of the splenic TCZ-specific TCRβ repertoires revealed that CD154 deficiency shifts the distribution of Vβ-Jβ genes after antigen exposure. This data led to the conclusion that costimulation via CD154:CD40 during the interaction of T cells with CD40-matured B cells contributes to the recruitment of T-cell clones into the immune response and thereby shapes the peripheral TCR repertoire.

## Introduction

The T-cell receptor (TCR) repertoire is shaped during negative, positive, and agonist selection in the thymus and by inter-clonal and intra-clonal competition in the periphery in adults after thymic involution. The latter is mainly triggered by homeostatic proliferation of naive T cells and by expansion of individual T-cell clonotypes after antigen exposure (1, 2). Clonal expansion and the resulting numbers of progeny depend on the strength of signals transmitted via the TCR upon ligation to its cognate peptide-MHC ligand. Thereby T cells with stronger TCR signaling generate bigger burst sizes (3–5). In addition to TCR signaling, the activation and lineage decision of CD4 T cells is regulated by costimulatory pathways. In particular, costimulation via CD154:CD40 that takes place during the interaction of antigen-activated T and B cells is critical for the differentiation of CD4 T cells into cytokine-producing effector T cells (6–8). The assumption that CD154 costimulation contributes to TCR signaling intensity leads to the hypothesis that it could provoke the enrichment or diminishment of individual T-cell clones during T-dependent humoral immune responses. Thus, the peripheral TCR repertoire could become narrower, shifted or alternatively broader due to CD154:CD40 costimulation supporting the clonal expansion of additional T-cell clonotypes that would otherwise be outnumbered by inter-clonal competition.

It has been shown previously that CD154 costimulation contributes to the TCR repertoire diversity during thymocyte development. Here, during the process of negative selection, CD154 deficiency permits the survival of T cells that bear specific Vβ segments, which are normally deleted in wild-type (WT) mice due to the recognition of superantigens presented in MHCII (9). Clear differences in the thymic T-cell repertoire have been described between WT and CD154-deficient mice, which were especially prominent in mice expressing the H-2E molecule (such as BALB/c mice) but also to a minor extent in mice expressing only the H-2A allele (such as C57BL/6 mice) (10–12). Whether CD154 costimulation affects the TCR repertoire during peripheral immune responses has not yet been clarified.

Here, we applied a high dose of sheep red blood cells (SRBCs) i.v. for induction of a strong local Th2 response in the spleen and isolated two individual T-cell zones (TCZ) by laser-microdissection (13) for analysis of the TCRβ repertoire by high-throughput sequencing. To compare the TCRβ repertoire between WT and CD154-deficient mice, we assessed typical features such as: (i) the number of TCRβ clonotypes, (ii) the percentage of identical TCRβ clonotypes between the groups, (iii) the frequency of individual TCRβ clonotypes, (iv) the length of their complementary determining region 3 (CDR3), and (v) the distribution of the V-J gene usage (14–16). Most of the differences were observed in both unimmunized and immunized mice, which clearly confirm the influence of CD154 costimulation during T-cell development in the thymus in C57BL/6 mice. However, the distribution of the V-J genes shifted differently after immunization in CD154-deficient mice compared to WT. These data demonstrate that CD154 costimulation influences the TCRβ repertoire not only during thymocyte development but also during T-cell differentiation in the periphery. Further studies are required to delineate whether targeting CD154 could be a therapeutic option to shape the TCR repertoire in a beneficial way in patients suffering from severe immune disorders.

## Materials and Methods

### Mice and Injections

8–12-week-old female C57BL/6J WT mice were obtained from Charles River Laboratories (Sulzfeld, Germany). CD154 (CD40L)-deficient mice (C57BL6; 129S2-Cd40lgtm1Imx/J; provided by D. Gray, Edinburgh, UK), and JHT (*gh-J^tm1Cgn^*, provided by Klaus Rajewsky, MDC Berlin) were bred in our animal facility (17, 18). Animal experiments were approved by local authorities of the Animal Care and Use Committee Kiel, Germany [V# 242-7224 122-1 (120-8/13) and (112-9/14)] and performed by certified personnel. A total of 10^9^ SRBCs (Labor Dr. Merk, Ochsenhausen, Germany) were prepared and injected into the tail veins as described (13). The spleens were removed before and 72 h after injection, snap frozen and stored at −80°C.

### Histological Analysis

Serial cryo-sections of spleens (10 µm thick for histology, 12 µm thick for laser-microdissection, and 14 µm thick for 3D reconstruction) were mounted on plain glass slides for histology and 3D model reconstruction or on membrane-covered slides (Palm Membrane Slides, PEN membrane, 1 mm; Carl Zeiss AG, Germany) for laser-microdissection. T- and B-cell compartments of spleens were analyzed by immunohistochemical staining with biotinylated mAbs against TCRβ and B220 (both from BD Biosciences). Alkaline phosphatase goat anti-rat IgG (Roth, Karlsruhe, Germany) and goat anti-hamster IgG (Abcam, Berlin, Germany) were used as respective secondary Ab. Alkaline phosphatase activity was visualized with Fast Red (BB Salt, Sigma-Aldrich Chemie, Steinheim, Germany). Proliferating cells were identified by staining with rat anti-mouse Ki-67 mAb as primary antibody (BioLegend, Koblenz, Germany) and biotinylated rabbit anti-rat IgG (Dako, Glostrup, Denmark) as secondary antibody as described (19). For laser-microdissection and subsequent RNA analysis, the staining with toluidine blue was performed as described (13).

### 3D Reconstruction

For each condition, a complete collection of serial cryo-sections from half a spleen was imaged by automatic scanning microscope and processed by For3D as described (20, 21) using a Miraxmidi slide scanner (Zeiss, Jena, Germany). ImageJ and homemade Matlab functions were used to render spleen sections into 3D. TCZ were segmented by filtering, thresholding and soothing the stack of spleen section images. Matlab was used to identify individual volumes of the 3D structures within the spleen as described (20, 21).

### Laser-Microdissection

To obtain sufficient concentration of TCR-specific RNA, it was important to carefully select the largest TCZ. A total of 10–15 serial sections had to be prepared for the isolation of whole large TCZ by laser-microdissection (Table 1; Figures 2A–C). Therefore, the two largest TCZs were chosen carefully and isolated using a pulsed UV laser (Palm Microbeam; Zeiss microImaging GmbH, Germany). To estimate the TCZ volumes, the isolated TCZ areas were determined by the Palm Microbeam software (Zeiss microImaging GmbH, Germany) and multiplied by the section thickness (12 µm) (Table 1). In order to prevent any degradation of RNA, the tissues were shock frozen immediately after isolation and not allowed to thaw during their preparation. All specimen were treated identically in order to exclude any biases between the mice.

**Table 1:** TCZ volumes, raw reads, total, and unique TCRβ sequences obtained from laser-captured splenic TCZ in wild-type (WT) and CD154-deficient (KO) mice.

	Mouse	TCZ	TCZ volume (×10^6^ µm^3^)	Raw reads (×10^6^)	Total TCRβ sequences (×10^6^)	Unique TCRβ clonotypes
WT naive	1	1	61	2.1	1.86	54652
		2	30	2.4	1.88	21016
	2	3	60	1.8	1.54	36625
		4	36	2.2	1.88	36196
	3	5	24	1.6	1.53	26190
		6	24	1.7	1.26	16477

	Mean ± SD		39.17 ± 17.12	1.97 ± 0.31	1.66 ± 0.26	31859 ± 13765

KO naive	1	1	33.6	1.3	0.8	16780
		2	32.4	1.9	1.02	13129
	2	3	43.2	1.5	1.0	10951
		4	48	1.6	1.4	23670
	3	5	33.6	1.0	0.8	25737
		6	33.6	1.0	0.8	24302

	Mean ± SD		37.40 ± 6.55	1.38 ± 0.35	0.97 ± 0.24	19095 ± 6316

WT SRBC	1	1	60	3.4	2.9	34926
		2	72	3.7	3.2	30141
	2	3	72	2.8	2.3	23253
		4	72	2.8	2.15	30200
	3	5	60	3.1	2.9	65306
		6	72	1.4	1.2	28173

	Mean ± SD		68.00 ± 6.20	2.87 ± 0.80	2.44 ± 0.73	35333 ± 15159

KO SRBC	1	1	40.8	1.7	0.7	28347
		2	38.4	1.3	0.8	12371
	2	3^a^	96	1.5	1.3	27909
		4^a^	93.6	1.6	1.5	43734
	3	5	36	1.3	1.2	25260
		6	36	1.3	0.8	12349

	Mean ± SD		56.80 ± 29.50	1.45 ± 0.18	1.05 ± 0.33	24995 ± 11749

*Two individual TCZ per spleen were isolated by laser-microdissection and subjected to deep sequencing*.

*^a^A total of 2–3 TCZ were pooled for one analysis*.

### Gene Expression Analysis

Five serial cryo-sections of spleens were prepared for isolation of the total RNA with the innuPREP RNA Mini Kit (Analytik Jena, Hildesheim, Germany). After reverse transcription, the cDNA and the respective primers were added to the Taq Man PCR Master Mix (Applied Biosystems) and amplified. The optimal primer concentrations used were 900 nM each for the forward and reverse primers and 200 nM for the TaqMan probe (Biomers, Ulm, Germany): IFN-γ (for: 5′GCAAGGCGAAAAAGGATGC, rev: 5′GACCACTCGGATGAGCTCATTG, probe: 5′TGCCAAGTTTGAGGTCAACAACCCACAG); IL-4 (for: 5′GAGACTCTTTCGGGCTTTTCG, rev: 5′AGGCTTTCCAGGAAGTCTTTCAG, probe: 5′CCTGGATTCATCGATAAGCTGCACCATG); and MLN51 (for: 5′CCAAGCCAGCCTTCATTCTTG, rev: 5′TAACGCTTAGCTCGACCACTCTG, probe: 5′CACGGGAACTTCGAGGTGTGCCTAAC). For signal detection, the ABI Prism 7000 sequence detector (Applied Biosystems, Darmstadt, Germany) was used. The amount of cDNA copies was normalized to the housekeeping gene MLN51 according to the 2**^ΔΔ^**^ct^ method (13, 22).

### Identification of TCRβ Clonotypes Within Splenic TCZ by High-Throughput Sequencing

The RNA from TCZ was isolated as described above. The preparation of cDNA and amplification of the antigen-binding site (CDR3β region) of the TCRβ chains were performed according to the manufacturer’s protocol (iRepertoire, patent 7999092, 2011, Huntsville, USA) and prepared for pair-end sequencing with the Illumina Miseq system as described (23). CDR3 identification, clonotype clustering, and correction of PCR and sequencing errors were performed using ClonoCalc wrapping MiTCR software according to the IMGT nomenclature (16, 24, 25). To avoid unpredictable PCR and sequencing errors, the default parameters (“eliminate these errors”) were used. Additionally, to avoid artificial diversity due to PCR errors, all TCRβ clonotype sequences that appeared only once were removed (on average 4% of all sequencing reads). To compare similarity or diversity among the groups we calculated the Jaccard Index. Therefore, we asked how many TCRβ clonotypes that exist in one TCZ would be present also in all other TCZ from the other mice (excluding the one TCZ from the same mouse). By doing this for each TCZ 12 values (Jaccard indices) for each group were determined (Table S1 in Supplementary Material). These 12 values were taken for statistical analysis (two-way repeated measures ANOVA with Tukey’s multiple comparison test). Further data analysis [frequency distribution (Figure 3), CDR3 length (Figure 4), principal component analysis (PCA) of V-J usage (Figure 5)] was performed after normalization of TCRβ clonotypes to the total number of TCRβ sequences (Table 1) using the R programming language, including the tcR package (15).

### Statistical Analysis

Statistical analyses were performed using GraphPad Prism 5.0 (GraphPad Software Inc., La Jolla, USA). Statistical significance was assessed by Kruskal–Wallis test, Mann–Whitney *U*-test, two-way repeated measures ANOVA with Tukey’s multiple comparison test, and multiple *t*-tests, one per row corrected for multiple comparisons with the Holms Sidak method. A *p* value of less than 0.05 was considered statistically significant.

## Results

### CD154 Costimulation Is Essential for CD4 T Helper Cell Differentiation into Th2 Cells and B-Cell Maturation

**Figure 1:**
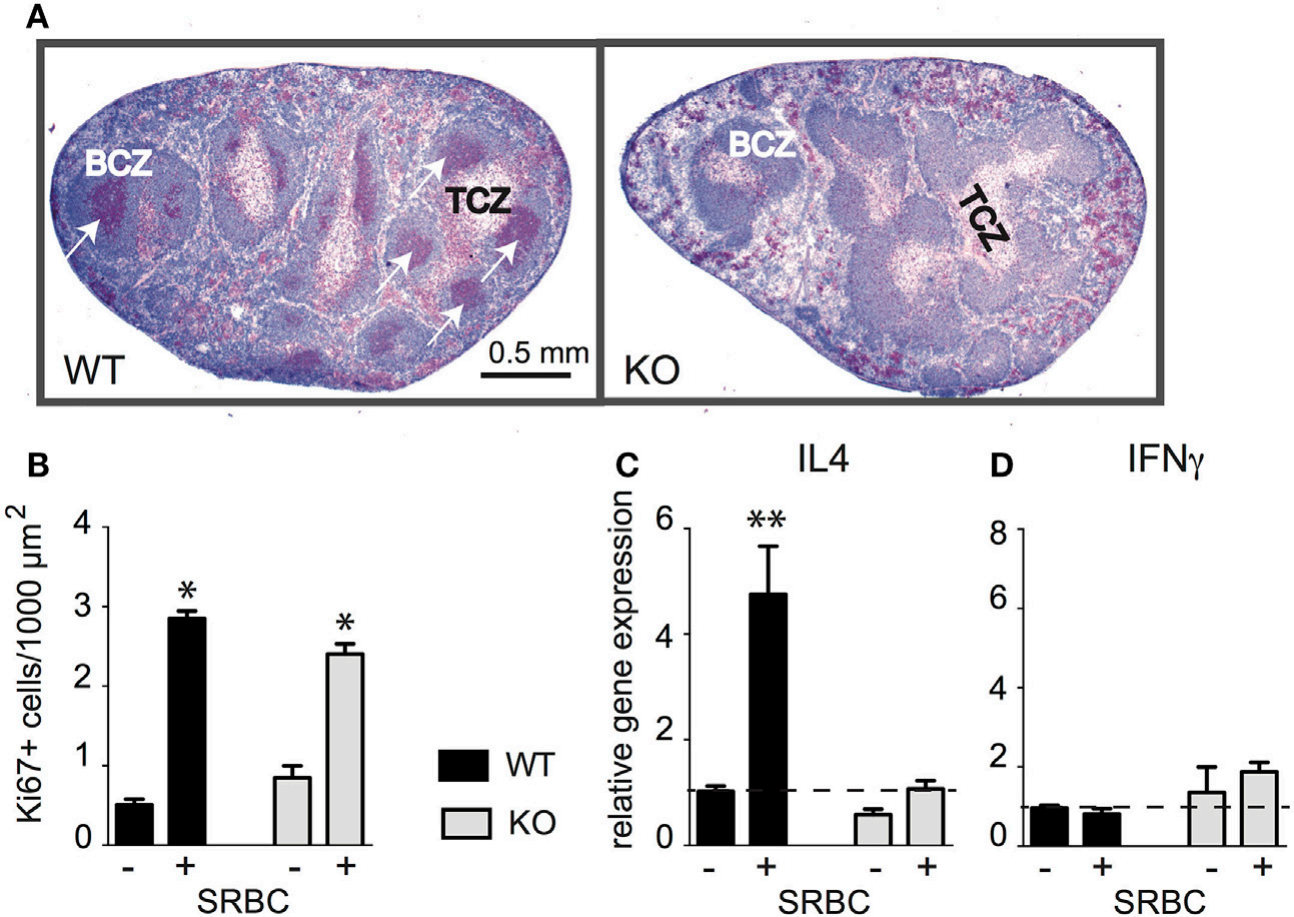
CD154 costimulation is essential for the Th2 differentiation of CD4 T cells and the formation of germinal centers (GC) but not for T-cell expansion. Wild-type (WT) and CD154-deficient (KO) mice were primed with 10^9^ sheep red blood cell (SRBC) intravenously. Splenic sections were stained for B cells (blue, B220) and proliferating cells (red, Ki-67+). **(A)** Proliferating cells in spleens from WT and CD154-deficient mice 10 days after injection of SRBC are shown. White arrows indicate GC in WT mice. **(B)** Proliferating cells (red, Ki-67+) were counted within the T-cell zones (TCZ) before and 3 days after injection of SRBC [*significant differences between the number of proliferating T cells compared to unchallenged mice; mean ± SEM (Kruskal–Wallis test), *n* = 3, *p* < 0.05]. **(C,D)** mRNA expression of IL-4 and IFNγ was analyzed by real-time RT-PCR and normalized to the housekeeping gene MLN51 before and 3 days after challenge with SRBC and displayed as *x*-fold increase compared to control spleens from non-injected mice. Dotted lines mark mean expression levels of controls [data show mean ± SEM (Kruskal–Wallis test), *n* = 6–7, **p < 0.01].

It has been shown previously that CD154 deficiency has bidirectional effects during T-dependent humoral immune responses: (i) it impairs the differentiation of CD4 T cells despite normal T-cell expansions and (ii) it abolishes germinal centers (GC) formation and affinity maturation of B cells (26–28). However, some reports demonstrated that primary GC could appear even under CD154-deficient conditions (29). To investigate whether a high dose of SRBC induces GC in CD154-deficient mice we monitored B-cell proliferation immunohistochemically 10 days after injection. GC were observed in WT mice but not in CD154-deficient mice (Figure 1A).

Next, we quantified proliferating T cells and determined respective mRNA expression levels of the Th1 cytokine IFNγ and the Th2 cytokine IL-4. At 3 days after injection, the peak of T-cell proliferation, we observed a three- to fivefold increase of proliferating cells in the TCZ of both groups (Figure 1B). In contrast, the IL-4 mRNA expression increased only in WT mice and was completely abolished in CD154-deficient mice (Figure 1C) whereas the expression of IFNγ did not change in either group (Figure 1D). To find out whether DC or B cells mediate the effects of CD154 costimulation additional experiments with B-cell-deficient (JHT) mice were performed. The result revealed that B cells are required for the induction of IL-4 mRNA expression (Figure S1 in Supplementary Material). The crucial role for B cells in this model was further supported by their increased expression of MHCII and their uptake of CFSE-labeled SRBC *in vivo* (Figures S2 and S3 in Supplementary Material). In conclusion, our data show that CD154 deficiency impairs GC formation and Th2 differentiation but has no effect on T-cell proliferation in response to SRBC.

### Laser-Microdissection Allows the Isolation of Complete TCZ

**Figure 2:**
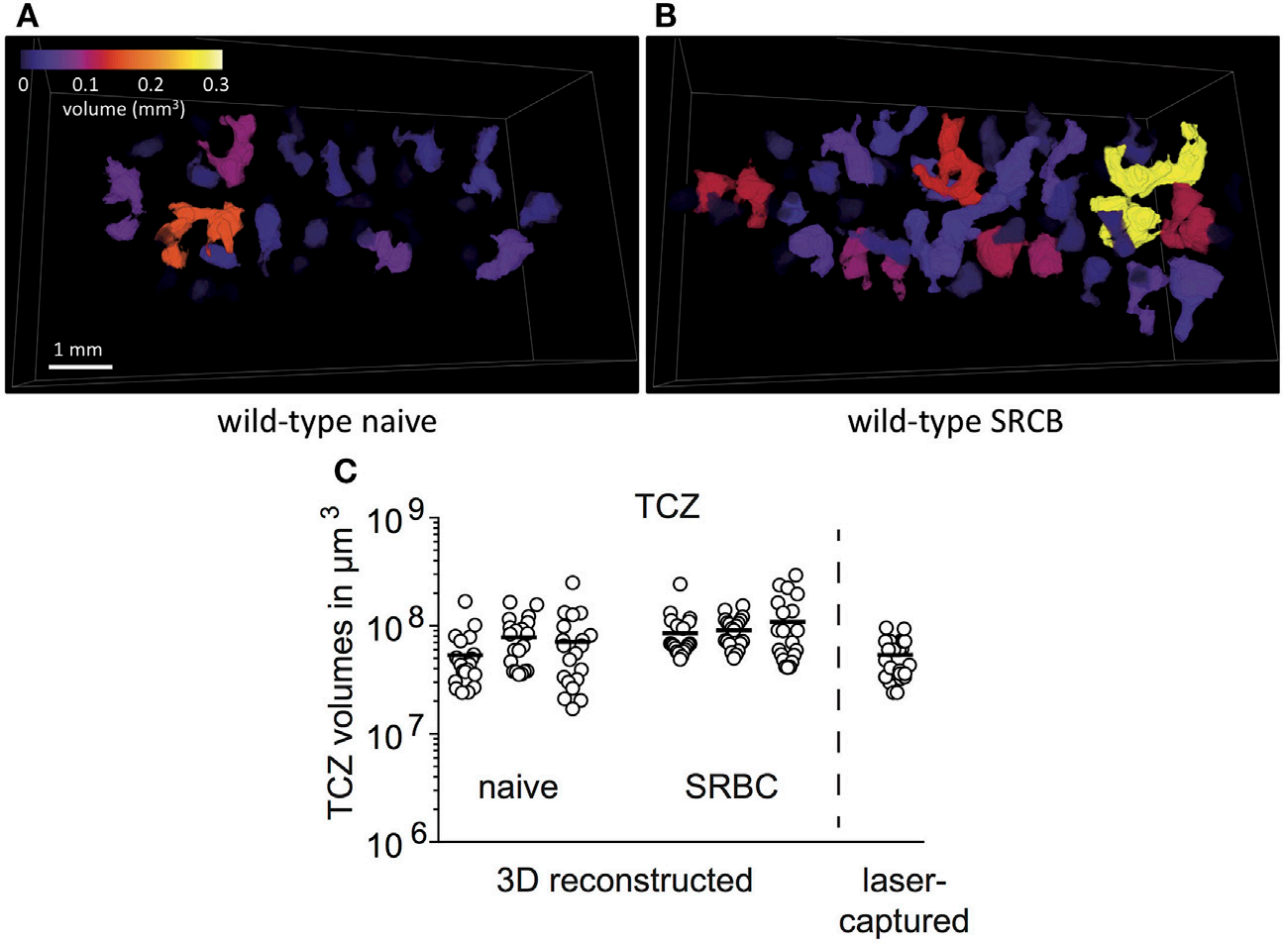
Laser-microdissection allows the isolation of entire T-cell zones (TCZ). A complete collection of sections of about half a spleen from wild-type mice was stained for B cells (blue, B220) and T cells (brown, TCRβ), imaged by automatic scanning microscope and 3D reconstructed by For3D software. **(A,B)** Color-coded individual TCZ from spleens of wild-type mice before **(A)** and after immunization **(B)** are shown. Please see Videos in Supplementary Material. **(C)** 3D reconstructed spleens were used to determine the volumes of the 20 largest TCZ with Matlab (*n* = 3, 20 TCZ per spleen). The volumes of three naive and three immunized spleens from wild-type mice are shown and compared to the volumes of TCZ that were harvested by laser-microdissection and estimated from 2D cryo-sections (*n* = 12, two TCZ per mice).

It is well known that TCZ are located around the splenic arteries in periarteriolar lymphoid sheaths (30). However, the organization of these structures in whole spleens is not well described. Most current data were obtained and extrapolated from two-dimensional tissue sections. Here, we performed a 3D reconstruction from half of the spleens (20, 21). Splenic TCZ appear as individual entities of highly diverse shape and size scattered throughout the spleen in transversal and longitudinal directions (Figure 2A; Video S1 in Supplementary Material). The volumes of the 20 largest TCZ range from 17 × 10^6^ to 290 × 10^6^ µm^3^ in naive and immunized spleens (Figure 2C). Due to the irregular shapes, it appears difficult to laser-capture a TCZ completely from two-dimensional cryo-sections. Therefore, only the two largest TCZ of one spleen were selected for isolation. Estimation of the laser-captured TCZ volumes revealed sizes of on average 53 ± 2 × 10^6^ µm^3^ (mean ± SD) (Table 1), which is in the range of an entire TCZ. In conclusion, through the use of a stack of serial sections, an almost complete TCZ can be harvested by laser-microdissection (Figure 2C).

### CD154 Deficiency Increases the TCR Diversity in Splenic TCZ

**Figure 3:**
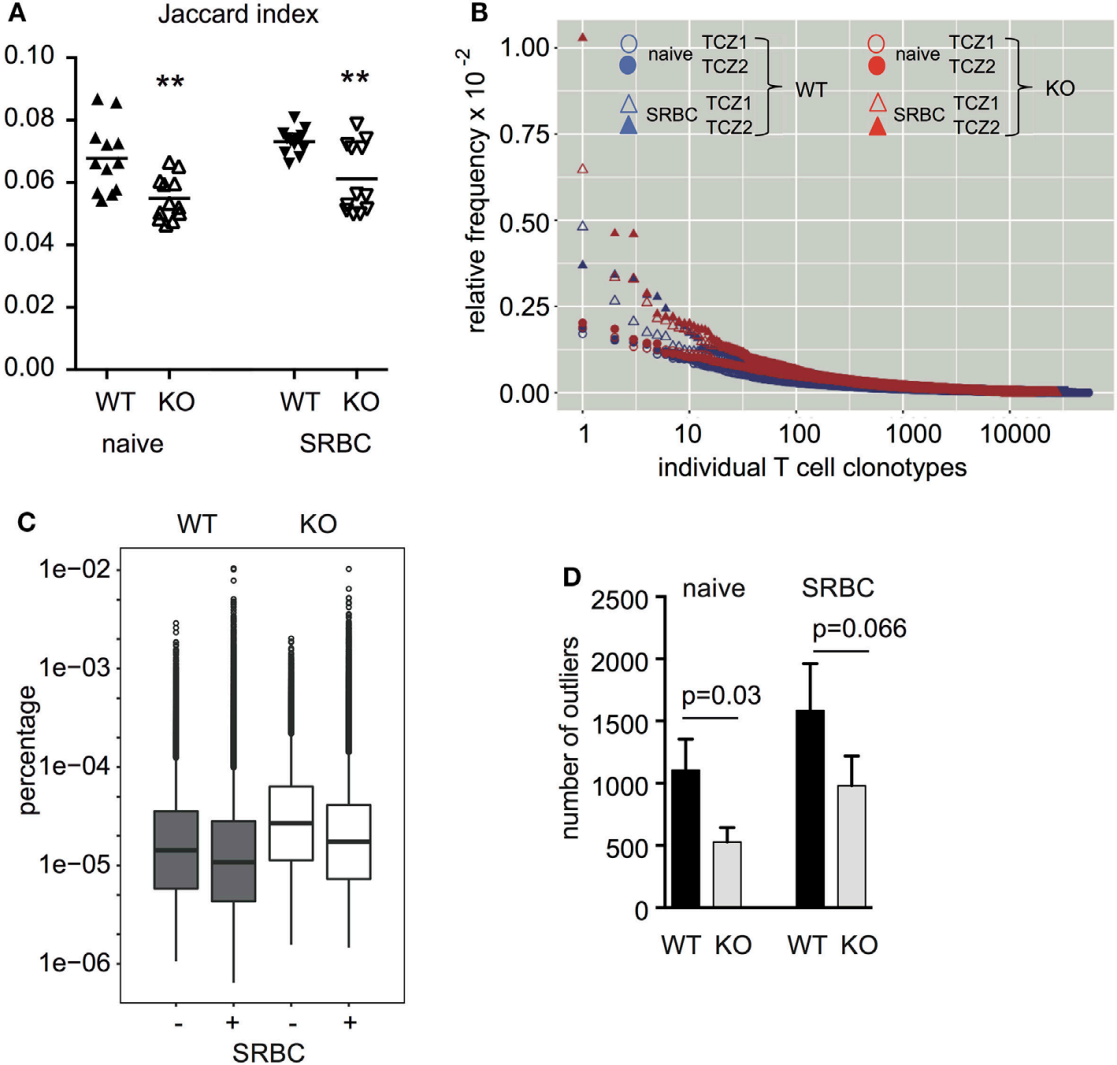
The diversity of TCRβ clonotypes is higher among the group of CD154-deficient mice compared to wild-type. Two TCZ per spleen of wild-type and CD154-deficient mice before and after immunization were laser-captured (two TCZ, *n* = 3 mice per group). TCRβ clonotypes were identified for each TCZ (six TCZ per group). **(A)** For every pair of TCZ within one group (excluding the TCZ of the same mouse) the Jaccard index, that is the number of TCRß clonotypes shared between the two TCZ relative to the total number of TCRß clonotypes occurring in the two TCZ together, is displayed (see Table S1 in Supplementary Material). In addition, the respective means are shown (**p ≤ 0.01, two-way repeated measures ANOVA with Tukey’s multiple comparison test). **(B)** The relative frequency of TCRβ clonotypes of two representative TCZ from one spleen per group [wild-type (blue) and CD154-deficient mice (red) before and after immunization] is shown. **(C)** Box plot analyses were performed to compare the frequency distribution of TCRβ clonotypes. Copy numbers relative to the number of all TCRβ sequences obtained are displayed (*n* = 3, two TCZ per spleen). Extremely high-frequent TCRβ clonotypes (outliers) that are outside an IQR of 3 are displayed as single dots. The following relative median clonotype frequencies were found: 14.3 × 10^−6^ for naive wild-type mice, 10.8 × 10^−6^ for wild-type mice after exposure to sheep red blood cell (SRBC), 26.87 × 10^−6^ for naive CD154-deficient mice, and 17.4 × 10^−6^ for CD154-deficient mice after exposure to SRBC. **(D)** Absolute numbers of high frequent outliers were compared between groups. Indicated are mean and standard error (*p* value is displayed for difference between indicated groups, Mann–Whitney *U*-test, *n* = 3, two TCZ per spleen).

Next, we isolated TCZ from WT and CD154-deficient mice, which were immunized or not. To exclude the possibility that CD154 deficiency influences the structure of the spleen and thereby the sizes and organization of the TCZ and B-cell zones (BCZ) a quantitative analysis of splenic compartments was performed (31). TCZ made about 40% and BCZ about 50% of the splenic area in both groups (Figure S4 in Supplementary Material). Due to the fact that no difference was observed regarding the TCZ and BCZ, we collected an identical area of TCZ from WT and CD154-deficient mice and analyzed their TCZ-TCRβ repertoire by high-throughput sequencing. We obtained between 0.8 and 1.88 × 10^6^ total TCRβ sequences for TCZ of naive spleens and from 0.7 to 3.2 × 10^6^ for TCZ of activated spleens, which contained between 10951 and 54652 unique TCRβ sequences (here referred to as TCRβ clonotypes) before immunization and from 12371 to 65306 after immunization, respectively, regardless whether the TCZ derived from WT or CD154-deficient mice (Table 1). The diversity occurring within each of the four groups (WT and CD154-deficient mice; unimmunized and immunized mice) was assessed as Jaccard index (Figure 3A; Table S1 in Supplementary Material). It provides a measure of similarity of samples and ranges from 0 to 1 as described in the method section (0, 100% different; 1, 100% identical). By contrast the Jaccard index is significantly lower in CD154-deficient mice compared to WT regardless of whether the mice were immunized or not. These data indicate that CD154 deficiency increases the number of distinct TCRβ clonotypes within the group of CD154-deficient mice. However, the Jaccard index does not differ significantly between unimmunized and immunized mice of either group, which indicates that CD154 deficiency influences the TCR diversity during thymic selection but not during the primary immunization with SRBC.

### CD154 Deficiency Marginally Reduces the Number of High Frequent Outliers

For comparison between the groups we elucidated the frequency of each individual TCRβ clonotype. Frequency plots were created in which each TCRβ clonotype is displayed as a single dot (x-axis) and arranged according to its relative frequency (y-axis) after normalization to the total number of TCRβ clonotypes in the respective TCZ. As shown in Figure 3B, a minority of TCRβ clonotypes appear at higher frequencies than the vast majority. Consistent with the fact that antigen-specific TCRβ clonotypes expand clonally after antigen exposure, the frequency of some high-expanded TCRβ clonotypes increases in both groups (Figure 3B; triangles compared to circles). For quantification, box plot analyses were performed. As Figure 3C shows, the distribution of copy numbers of individual TCRβ clonotypes is far from a Gaussian distribution in all four groups. Between 500 and 1,500 of the high frequent TCRβ clonotypes were identified as outliers irrespective of whether 1.5 (data not shown) or 3 was chosen as the interquartile range (Figures 3C,D). Enumeration of TCRβ outliers revealed that CD154 deficiency significantly reduces the accumulation of outliers compared to WT mice (Figure 3D). This reduction is found in both unimmunized and immunized mice, which indicates that CD154 costimulation regulates the number of outliers mainly during thymic selection but does not influence the expansion of TCRβ clonotypes during the immune response to SRBC.

### CD154 Deficiency Selects T Cells With Shorter CDR3 Regions

**Figure 4:**
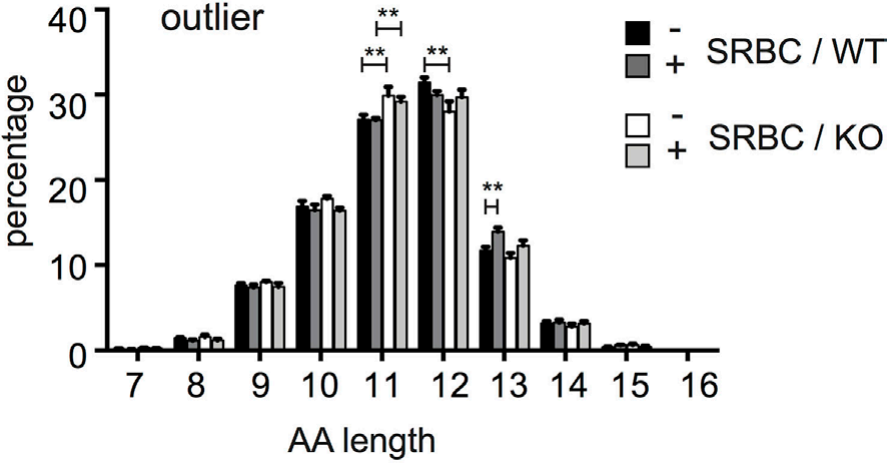
TCRβ outliers of CD154-deficient mice have shorter complementary determining region 3 (CDR3) regions. Relative length distribution of CDR3 regions of the high-frequent TCRβ outliers as described in Figures 3C,D were compared between wild-type and CD154-deficient mice before and after immunization. Mean ± SEM is shown, and bars above columns indicate significant differences between groups multiple *t*-tests, one per row corrected for multiple comparisons with the Holms Sidak method; **p ≤ 0.01.

To further compare the TCZ-TCR repertoires between WT and CD154-deficient mice, we compared the length of their antigen-binding sequence, the CDR3 region. Given that the outliers better survive the inter-clonal competition and expand preferentially during thymic and peripheral selection, we assumed that they might represent the TCRβ clonotypes that are most affected by CD154 deficiency and therefore compared the length of the CDR3 region exclusively between the outliers of both groups (see Figures 3C,D). However, the CDR3 length of TCRβ outliers from CD154-deficient mice is shorter compared to WT mice. This applies in particular for the TCRβ clonotypes with a CDR3 length of 11 and 12 amino acids (Figure 4). For example, about 28% of the high frequent TCRβ outliers from WT mice have a CDR3 length of 11 AA and about 31.4% from CD154-deficient mice. In turn, a higher percentage (29.9%) of the high frequent outliers from WT mice have a length of 12 AA compared to only 27.2% in CD154-deficient mice (first and third columns, Figure 4). These shifts were found in both unimmunized and immunized mice, which indicate that the enrichment of TCRβ clonotypes with shorter CDR3 regions in CD154-deficient mice takes place during thymocyte development. However, marginal shifts in the CDR3 length due to the immune response to SRBC were found in WT outliers with a CDR3 length of 13 amino acids but not in CD154-deficient mice (Figure 4). The CDR3 length of the low or medium abundant T-cell clonotypes did not differ (data not shown).

### CD154 Signaling Controls the Selection of TCRβ Clonotypes During Immunization

**Figure 5:**
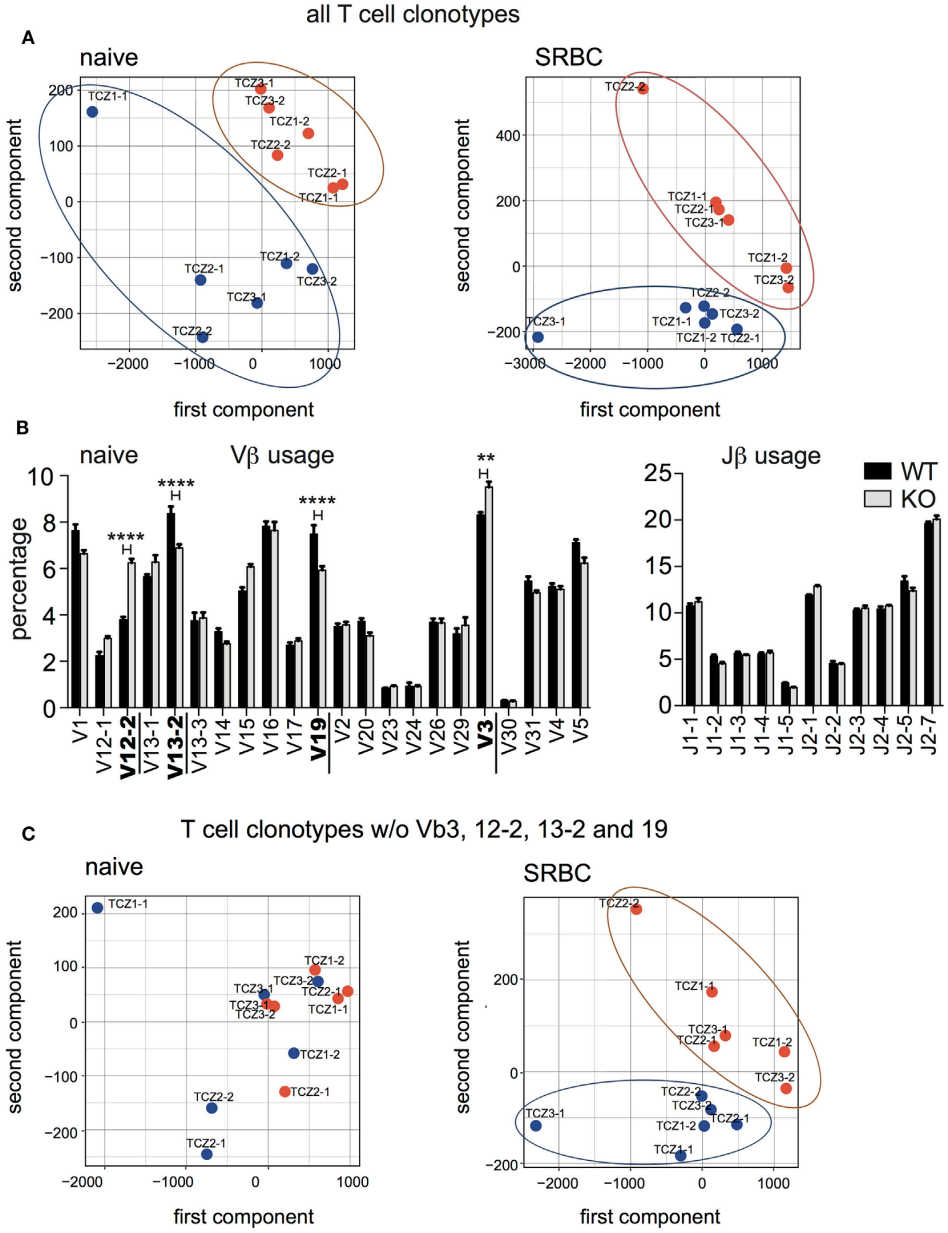
V-J gene usage shifts between wild-type and CD154-deficient mice in response to sheep red blood cell (SRBC). **(A)** Principal component analysis (PCA) compares V-J gene usage of TCRβ clonotypes in individual T-cell zones (TCZ) between wild-type (blue dots) and CD154-deficient mice (red dots) before (left panel) and after immunization (right panel). **(B)** Relative frequencies of Vβ (left) and Jβ (right) genes were compared between naive wild-type and CD154-deficient mice. Indicated are means ± SEM. Vβ genes with significantly different usage are displayed in bold (two-way repeated measures ANOVA with Tukey’s multiple comparison test, **p ≤ 0.01 for Vβ3, ****p ≤ 0.0001 for Vβ12-2, 13-2, and 19). **(C)** PCA without Vβ3, Vβ12-2, Vβ13-2, and Vβ19 of V-J gene usage of TCRβ clonotypes in TCZ between wild-type and CD154-deficient mice before (left panel) and after immunization (right panel).

We compared the V-J gene usage between splenic TCZ-TCRβ clonotypes from WT and CD154-deficient mice by PCA (Figure 5A). The TCRβ clonotypes from both groups localize in distinct clusters. The result that the TCRβ clonotypes of WT and CD154-deficient mice remain separated after immunization reveals that shifts in V-J gene usage are induced during thymic selection, which has been described before (Figure 5A, left and right panel) (12).

In more detail, distribution analysis of the individual Vβ and Jβ gene segments revealed that WT mice have significantly less TCRβ clonotypes with TCR expressing Vβ12-2 and Vβ3. Conversely, TCRβ clonotypes carrying the Vβ13-2 and Vβ19 genes were significantly enriched (Figure 5B). No significant difference in the Jβ gene usage was observed. After removal of all 4 Vβ genes (Vβ12-2, Vβ13-2, Vβ19, and Vβ3), which are sensitive to CD154 signaling in naive mice, the two groups are not separated anymore. (Figure 5C, left). However, distinct clusters of TCRβ clonotypes of WT and CD154-deficient mice reappear after exposure to SRBC (Figure 5C, right). Here, a significantly higher percentage of Vβ15 and Jβ2-1 was found in CD154-deficient mice compared to WT (data not shown). This data indicates that the lack of CD154 costimulation leads to an accumulation of TCRβ clonotypes expressing Vβ15 and Jβ2-1 segments whereas the presence of CD154 costimulation supports a more uniform distribution of the TCRβ clonotypes in regard to their V-J gene usage during the response to SRBC.

## Discussion

A highly diverse peripheral TCR repertoire is a prerequisite for successful responses to infections or vaccinations (32). A decreased diversity has been linked to chronic infections (33), aging (2) and several autoimmune diseases (34, 35). The factors that form the peripheral TCR repertoire during immune responses as well as during lifetime are poorly defined. The current available data allow catching a first glimpse only on the extreme diversity of the TCR repertoire and its regulation. Here, we asked whether T-cell costimulation during peripheral immune responses contributes to the diversity of the TCRβ repertoire.

CD154, a member of the TNFR superfamily, is transiently expressed on antigen-activated T cells. Its ligand CD40 is found on antigen-presenting cells such as dendritic cells, B cells and macrophages but also on thymic epithelial cells. CD154 is a key molecule for T-cell survival in the thymus and CD4 T-cell differentiation in the periphery (36, 37). The effects of CD154 deficiency on the TCR repertoire of thymocytes have been described previously. In these studies monoclonal antibodies directed against certain Vβ segments from thymocytes of BALB/c mice have been used (11, 12).

Our study provides for the first time high-quality data on global TCR repertoires of the spleen as a central peripheral secondary lymphoid organ in unimmunized and immunized WT and CD154-deficient mice. To focus on the local TCR repertoire we isolated two TCZ per spleen by laser-microdissection. Besides the advantage that the local distribution of T-cell clonotypes remains undisturbed, this approach avoids any potential loss of cells during isolation procedures. This is of importance because a significant number of splenic T cells get lost by conventional isolation techniques (38). One could assume that the TCR repertoires could be biased because especially the highly activated T cells are prone to undergo apoptosis during the isolation steps compared to their resting counterparts. To find out it will be necessary to compare the TCR repertoires *in vivo* within the tissues and *in vitro* after isolation.

Analysis of the diversity of the TCZ-TCRβ repertoire revealed that the group of CD154-deficient mice shares less TCRβ clonotypes than the WT group, which indicates that splenic TCZ of CD154-deficient mice harbor a higher number of different TCRβ clonotypes (Figure 3A). This observation is consistent with the impaired deletion of T-cell clonotypes during thymic negative selection in CD154-deficient mice (10, 11).

In general, our data reveal that CD154 costimulation has a strong impact on the selection of T-cell clones during T-cell development in the thymus. In the thymus, an increased TCR signaling strength during CD154 costimulation leads to the induction of apoptosis of those T-cell clones that bind with the strongest avidity to self-peptide MHCII complexes during the negative and agonist selection process. It is, therefore, expected that more T cells survive and diversity increases under CD154-deficient conditions. This higher diversity comes along with fewer outliers (Figure 3D), small shifts toward a shorter CDR3 region (Figure 4) and a higher number of T-cell clones that express Vβ12.2 and Vβ3 in CD154-deficient mice (Figure 5).

The situation is different during peripheral immune responses. Here, an increased TCR signaling strength should increase the progeny of the antigen-specific T-cell clones. Unexpectedly, our findings do not confirm this assumption. By analyzing the global TCZ-TCR repertoires, we find that CD154 costimulation has no effect on the diversity and the number of outliers during the primary immune response to SRBC. This finding might be explained by the fact that during T-cell development in the thymus each thymocyte is activated to express CD154 and selected for binding to CD40, whereas during peripheral immune responses only those T cells that are specific for the antigen have the chance to contact cognate CD40-matured B cells. It could be that the effects of CD154 costimulation would become more obvious if analysis was restricted to the antigen-specific CD4 T cells only, for example with the use of MHCII tetramers (4), instead of the bulk analysis performed here. However, recent studies indicated that the TCR sequences of antigen-specific T cells are extremely divers even in genetically identical and cage-matched mice. Many different T-cell clonotypes of both high and low frequency, rather than the dominant expansion of a few dominating antigen-specific clones, contribute to the immune response (39). In addition, it could be that the effects of CD154 costimulation could become stronger after repeated immunization with the same antigen. Here, we chose SRBC for immunization because it has multiple epitopes and induces CD4 T-dependent humoral immune responses without the need for adjuvants (40, 41). We assumed that SRBC would be recognized not only by the toll-like receptors of professional antigen-presenting cells due to their RNA content (42) but also by B cells due to the carbohydrate structures present on the surface of each red blood cell. The fact that the activation of B cells is critical for CD4 T-cell differentiation into Th2 cells was shown in B-cell-deficient mice (Figures S1–S3 in Supplementary Material). In addition, it has been demonstrated previously by administration of low or high doses of SRBC (13, 43). The major role of CD40–CD154 signaling during T–B interaction is further underlined by previous *in vitro* studies that demonstrated that this lack of IL-4 expression is not caused by an intrinsic inability of CD154-deficient T cells to express IL-4 (6). The level of TCR signaling strength might be increased under *in vitro* conditions due to a boosted peptide:MHCII density or a shifted ratio of antigen-presenting cells to T cells compared to the *in vivo* situation, which could enable the expression of IL-4 even without CD154 costimulation (6, 44). However, even though SRBC induced a strong polyclonal T-cell response in WT and CD154-deficient mice (Figure 1B) *in vivo*, the expression of IL-4 was impaired in CD154-deficient mice (Figure 1C). In addition, the obtained TCRβ repertoire data revealed that CD154 deficiency had no effect on the diversity and the number of outliers. Further studies are required to address the differences between TCR repertoire data obtained by analysis of antigen-specific T cells versus: (i) bulk analysis, (ii) the impact of secondary and tertiary immunizations, and (iii) the role of the nature of the antigen.

Unexpectedly, we detected shifts in the V-J gene usage due to the immune response to SRBC. This difference becomes visible only after exclusion of those V-J genes that were affected by CD154 deficiency during the thymic selection process. The question arises: what causes the distinct enrichment of T-cell clones according to their V-J genes? In the thymus the shifts in V gene usage have been linked to the expression of superantigens such as mouse mammary tumor virus, which are recognized predominantly by TCR expressing specific Vβ segments (9). Accordingly the shifts in V-J gene usage during the peripheral immune response could be due to differences in the presentation of SRBC-specific epitopes under CD154-deficient conditions. One possible explanation that would support this scenario is the finding that the costimulatory signals CD28:CD80/86 and CD154:CD40, which are crucial for the differentiation of CD4 T cells, precede as segregated events on distinct cells (8). This data leads to the assumption that B cells mount their own antigen-specific response, which is different from that of DC, and thereby recruit their own T-cell clones into the immune responses. Thus, in the case of CD154 deficiency, which impairs the interaction of CD4 T cells with B cells and prevents the antigen presentation by B cells, those T-cell clones that were activated preferentially by dendritic cells would accumulate more compared to the WT. Conversely, in WT mice with an intact antigen presentation by B cells, those CD4 T cells that were preferentially activated by dendritic cells would have to compete for interaction with CD40-matured B cells, which would clearly impact the composition of the individual T-cell clonotypes. A specific modulation of the B-cell response could therefore be a promising target for the modulation of the CD4 TCR repertoires.

Alternatively, the observed shifts in V-J segment usage could be explained by distinct migration behaviors. The lack of CD154:CD40 signaling during CD4 T-B interaction could prevent the migration into the BCZ of CD4 T cells that express the V-J segments that recognize SRBC-specific epitopes. To further clarify the effects of peripheral CD154 signaling it would be preferable to use CD154 conditional knockout mice. In addition, it will be interesting to find out whether CD40-deficient mice show a similar phenotype as observed under CD154-deficient conditions. CD40 is expressed on thymic epithelial cells before birth whereas CD154 expression was found only in neonatal mice (45). One could speculate that CD40-deficient mice would have a more skewed TCR repertoire than CD154-deficient mice.

In summary, in this study we provide evidence that CD154 signaling controls the selection of TCR clonotypes during a T-dependent humoral immune response. Further studies are required to investigate whether a variation of CD154 signaling could be used as a therapeutic option to modulate the TCR repertoire in a controlled manner. This could help to improve vaccines, treat autoimmune conditions, or prevent rejections after organ transplants. Due to this fundamental role of CD154 in adaptive immunity, CD154 signaling pharmacology for transplantation medicine and the treatment of autoimmune disorders is already being subjected to clinical trial (46, 47).

## Ethics Statement

Animal experiments were approved by local authorities of the Animal Care and Use Committee Kiel, Germany [V# 242-7224 122-1 (120-8/13) and (112-9/14)] and performed by certified personnel.

## Author Contributions

AF designed experiments and analyzed data. SeK designed and performed experiments. AS performed experiments and analyzed data. CN, SaK, SM, and KKe analyzed data and contributed to manuscript writing, JW designed parts of the study and contributed to manuscript writing. KKa directed the study and wrote the manuscript.

## Conflict of Interest Statement

The authors declare that the research was conducted in the absence of any commercial or financial relationships that could be construed as a potential conflict of interest.

## Abbreviations

BCZ, B-cell zones; CDR3, complementary determining region 3; GC, germinal centers; SRBCs, sheep red blood cells; TCR, T-cell receptor; TCZ, T-cell zones.
