# Supplementary material for: CD154 Costimulation Shifts the Local T-Cell Receptor Repertoire Not Only During Thymic Selection but Also During Peripheral T-Dependent Humoral Immune Responses
Source: Front Immunol. 2018 May 17;9:1019. doi: 10.3389/fimmu.2018.01019 (PMC5966529; doi:10.3389/fimmu.2018.01019)
Supplement: Supplementary file 1 [file Image_1.PDF]

Supplemental Figure 1

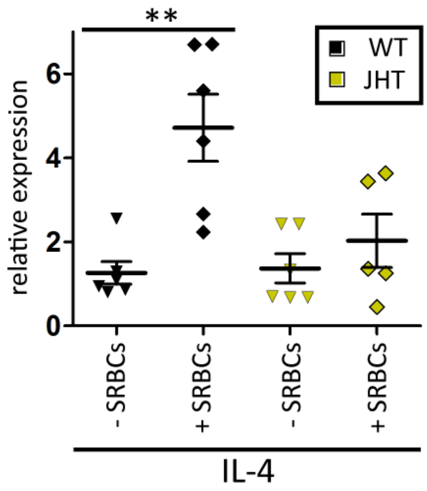

Figure S1. B cells are required for induction of IL4 expressing Th2 cells. Wild-type and B-cell deficient JHT mice were primed intravenously with SRBC. Relative mRNA expression of IL-4 in the spleen compared to the internal control on day 3 post immunization with PBS or SRBC. Horizontal lines represent mean ±SEM (Kruskal-Wallis test), n=5-6, **$p<0.01$.
