# Supplementary material for: CD154 Costimulation Shifts the Local T-Cell Receptor Repertoire Not Only During Thymic Selection but Also During Peripheral T-Dependent Humoral Immune Responses
Source: Front Immunol. 2018 May 17;9:1019. doi: 10.3389/fimmu.2018.01019 (PMC5966529; doi:10.3389/fimmu.2018.01019)
Supplement: Supplementary file 2 [file Image_2.PDF]

Supplemental Figure 2

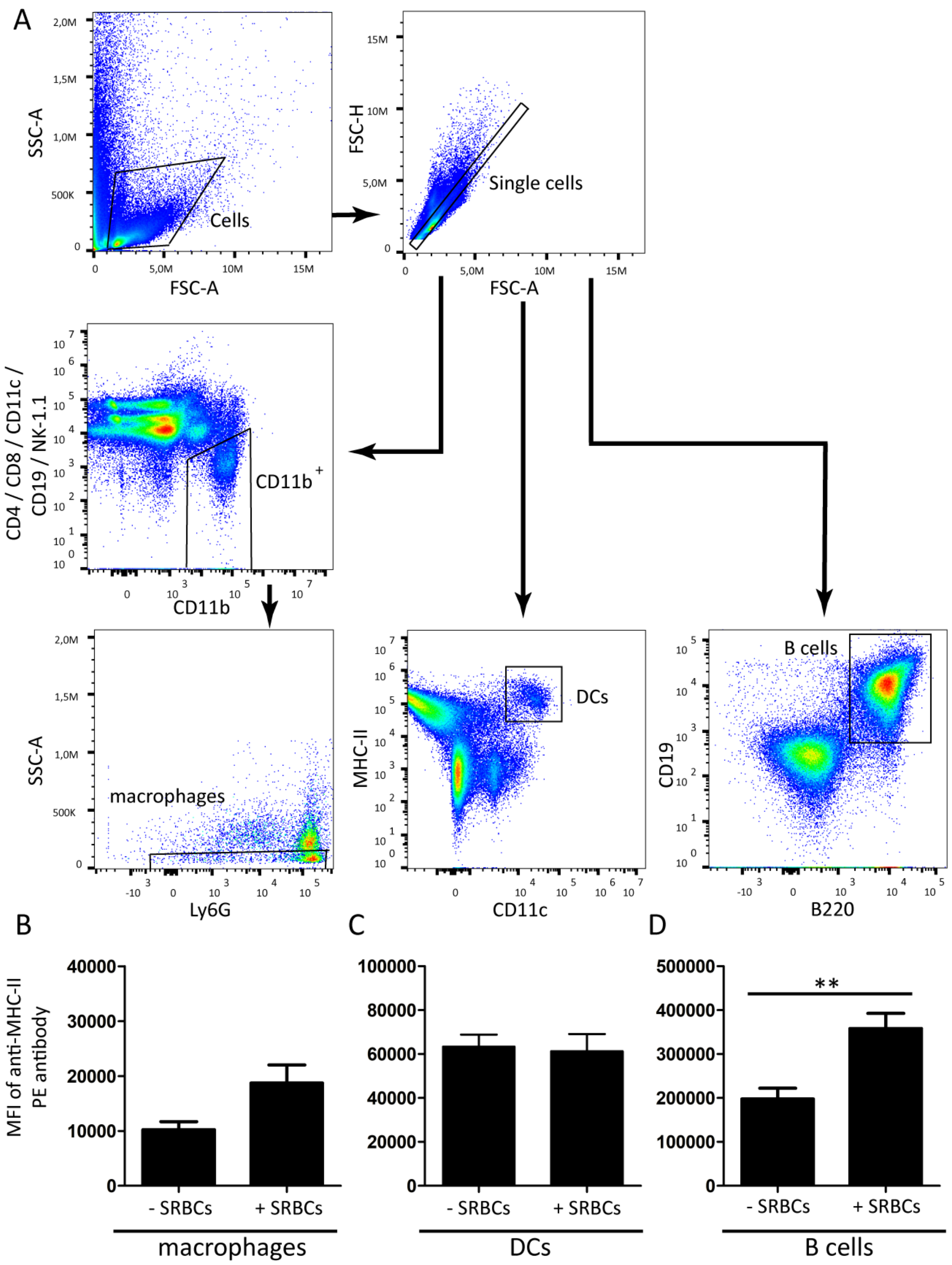

Figure S2. SRBC immunization increases MHC-II density on B cells. Mice were primed intravenously with SRBC. (A) Gating strategy and (B, C, D) MHC-II on the surface of splenic macrophages (CD11b+, Ly6Glow, SSClow) (B), DCs (CD11c+ Gate) (C) and B cells (CD19+, B220+) (D), 3 days post immunization with PBS or SRBCs (HD). Bars represent mean ±SEM, n=6, $^{*}p<0.05$, $^{**}p<0.01$ (Kruskal-Wallis test). The following antibodies were used: CD11b-FITC (M1/70, rat IgG2a), CD11c-APC (N418, Armenien hamster IgG), Ly6G-PE-Cy7 (ZB6-8C5, rat IgG2b), MHC-II-PE (M5/114.15.2, Rat IgG2b), B220-PerCP-Cy5.5 (RA3-6B2, rat IgG2a) (all obtained from eBioscience) and CD19-Alexa Flour 647 (1D3, Rat IgG2a) from BD Bioscience.
