# Supplementary material for: CD154 Costimulation Shifts the Local T-Cell Receptor Repertoire Not Only During Thymic Selection but Also During Peripheral T-Dependent Humoral Immune Responses
Source: Front Immunol. 2018 May 17;9:1019. doi: 10.3389/fimmu.2018.01019 (PMC5966529; doi:10.3389/fimmu.2018.01019)
Supplement: Supplementary file 3 [file Image_3.PDF]

Supplemental Figure 3

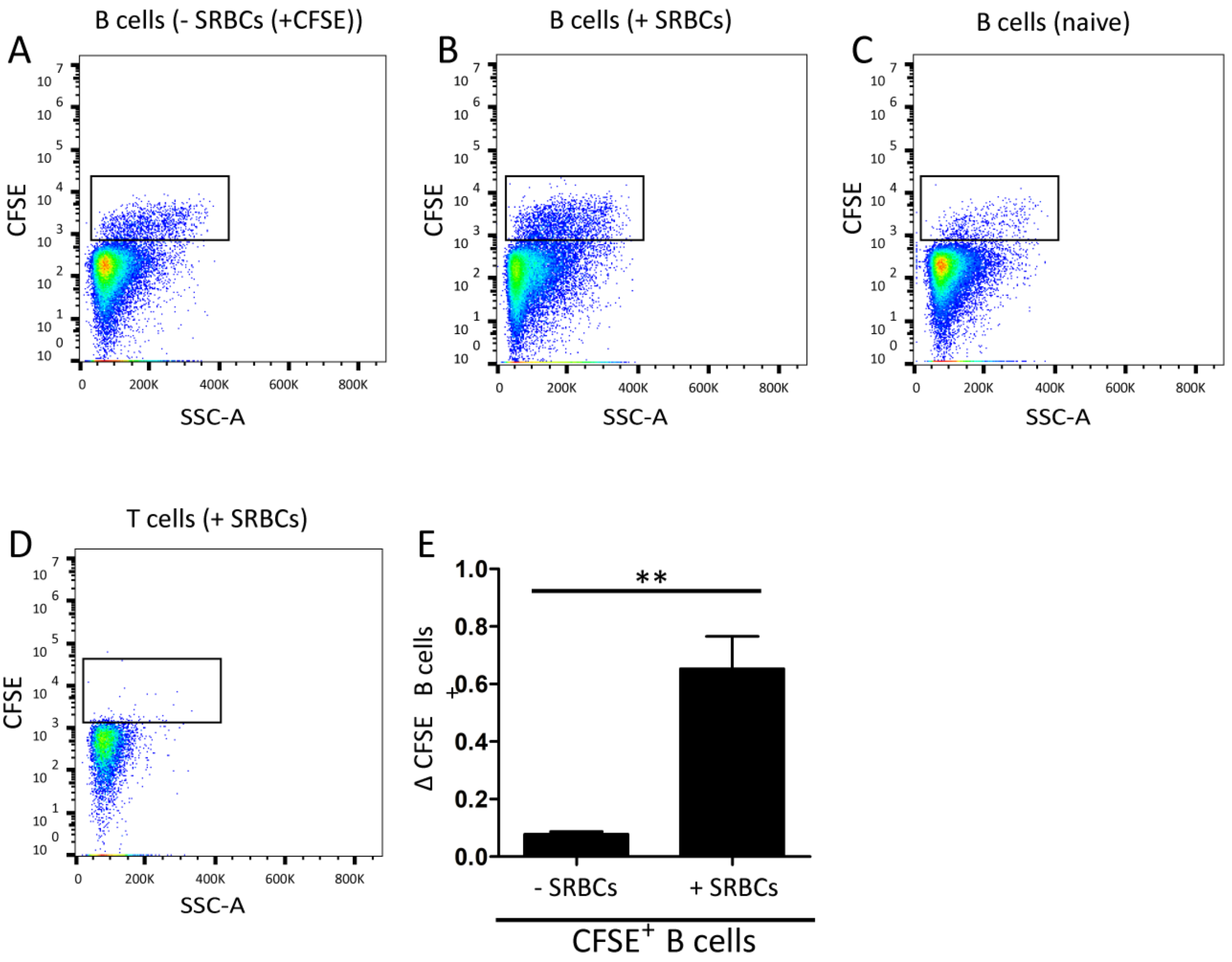

Figure S3. CFSE labeled SRBCs are taken up by B cells in vivo. CFSE-labeled SRBC were injected intravenously. Flow cytometric measurement of CFSE positive B cells (CD19+, B220+) (A, B, C) and T cells (TCRβ+, CD4+) (D) as control, 3 hours after different immunizations. CFSE+ B cells after PBS (ctrl) or SRBC immunization were quantified as the difference to naïve B cells (to exclude auto-fluorescence) and T cells (to exclude unspecific surface CFSE) (F). Bars represent mean ±SEM (Kruskal-Wallis test), n=6, **p<0.01.

The following antibodies were used: B220-PerCP-Cy5.5 (RA3-6B2, rat IgG2a) and CD4-APC (GK1.5, rat IgG2a) from eBioscience; CD19-Alexa Flour 647 (1D3, Rat IgG2a) and TCRβ (H57-597, Armenian hamster IgG2) from BD Bioscience.
