# Supplementary material for: CD154 Costimulation Shifts the Local T-Cell Receptor Repertoire Not Only During Thymic Selection but Also During Peripheral T-Dependent Humoral Immune Responses
Source: Front Immunol. 2018 May 17;9:1019. doi: 10.3389/fimmu.2018.01019 (PMC5966529; doi:10.3389/fimmu.2018.01019)
Supplement: Supplementary file 4 [file Image_4.PDF]

Supplemental Figure 4

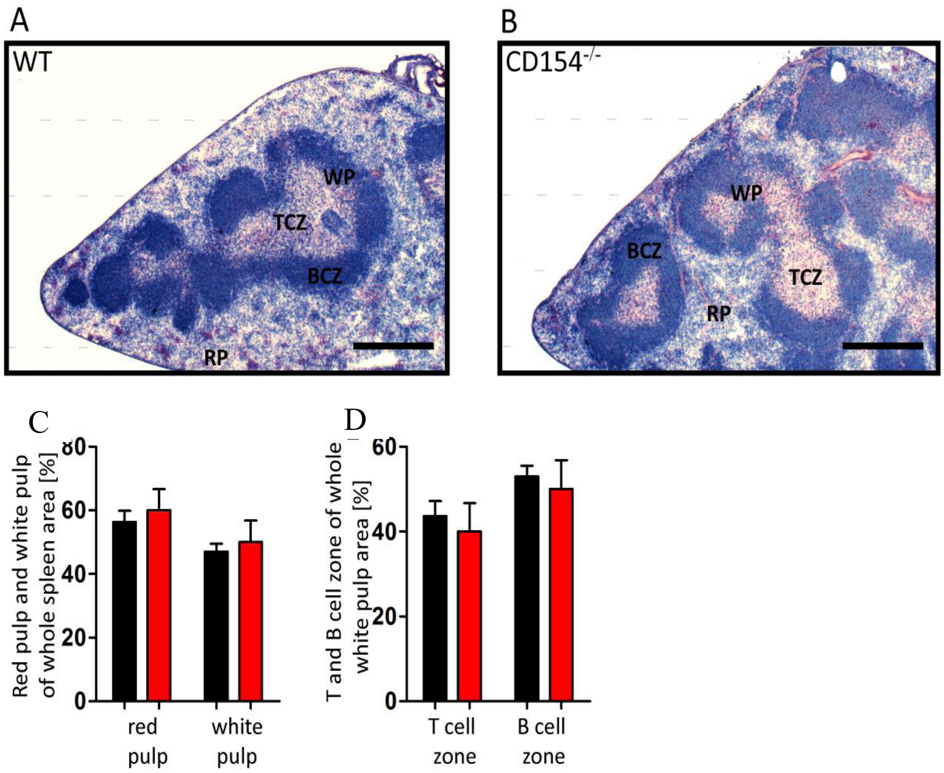

Figure S4: CD154 deficiency does not influence the structure of the spleen. Spleens (naïve WT (A) and CD154-/- (B) mice) were immunohistochemically stained with anti-Ki-67 (red, Poly4054, Goat IgG, Biolegend) and anti-B220 (blue, RA3-6B2, Rat IgG2a, BD Bioscience), scale bar represents 500 µm. The sizes of red pulp (RP) and white pulp (WP) (C) and T and B cell zone (D) were quantitatively assessed. Bars represent mean ±SEM (Mann-Whitney test), n=3.
