# Supplementary material for: CD154 Costimulation Shifts the Local T-Cell Receptor Repertoire Not Only During Thymic Selection but Also During Peripheral T-Dependent Humoral Immune Responses
Source: Front Immunol. 2018 May 17;9:1019. doi: 10.3389/fimmu.2018.01019 (PMC5966529; doi:10.3389/fimmu.2018.01019)
Supplement: Supplementary file 5 [file Table_1.PDF]

Supplemental table 1
The Jaccard index was calculated by assessing the number of TCRß clonotypes that were shared between every two TCZ of one group and dividing these number by the total number of TCRß clonotypes occurring in either of the two TCZ. Corresponding data and statistics are shown in Figure 3A.

sample1	sample2	wt/naive	wt/SRBC	cd154ko/naive	cd154ko/SRBC
TCZ1 mouse 1	TCZ1 mouse 2	0.087	0.071	0.046	0.072
TCZ1 mouse 1	TCZ2 mouse 2	0.086	0.076	0.050	0.079
TCZ1 mouse 1	TCZ1 mouse 3	0.074	0.081	0.053	0.071
TCZ1 mouse 1	TCZ2 mouse 3	0.056	0.074	0.052	0.051
TCZ2 mouse 1	TCZ1 mouse 2	0.066	0.070	0.050	0.056
TCZ2 mouse 1	TCZ2 mouse 2	0.066	0.073	0.060	0.053
TCZ2 mouse 1	TCZ1 mouse 3	0.064	0.075	0.060	0.056
TCZ2 mouse 1	TCZ2 mouse 3	0.054	0.072	0.059	0.050
TCZ1 mouse 2	TCZ1 mouse 3	0.073	0.066	0.048	0.071
TCZ1 mouse 2	TCZ2 mouse 3	0.058	0.068	0.048	0.052
TCZ2 mouse 2	TCZ1 mouse 3	0.072	0.078	0.067	0.074
TCZ2 mouse 2	TCZ2 mouse 3	0.057	0.074	0.065	0.050
